# Reforming the World Health Assembly

**DOI:** 10.1136/bmjgh-2020-002570

**Published:** 2020-05-15

**Authors:** Rachel Irwin

**Affiliations:** Arts and Cultural Sciences, Lund University, Lund, Sweden

**Keywords:** health policy, public health

The recent announcement to hold the 73rd World Health Assembly (WHA) in a virtual de minimis session was expected. Due to the ongoing COVID-19 pandemic, restrictions on travel, public gatherings and other aspects of daily life remain in force in many countries. The question of a digital WHA was already raised during the WHO Emergencies Press Conference on 6 March, in light of the World Bank’s decision to hold their Spring Meetings virtually.^1^

Aside from the formal decisions that are taken at the WHA, the event serves a significant social function with global health.^2^ There is web of symbiotic relationships between the formal proceedings and the array of informal and semiformal activities, such as technical briefings, side events, dinners, receptions and spontaneous meetings in the corridors of the Palais de Nations. These in-person interactions will be severely missed. However, the virtual 73rd WHA also offers an opportunity to consider how the WHA can change, and I suggest three points for consideration.

First, does the event need to be attended by over 4000 people? According to Article 11 of the WHO’s Constitution, each Member State can have no more than three delegates, but these may be accompanied by alternatives and advisors. In practice, there is a range of delegation sizes. For instance, at the 72nd WHA in 2019, Bulgaria had 5, the Democratic Republic of the Congo had 15, Sweden had 28 and the USA had 50 delegates, respectively.^3^ The omnipresent climate concerns posed by large delegations flying to Geneva is inconsistent for work meant to contribute to sustainable development.^4^ Live streaming is imperfect: there are concerns over security, access to reliable internet and the question of balancing time zones. However, a virtual meeting would allow for civil servants to follow the proceedings from home, thus reducing the climate burden.

Instead of sending large delegations, Member States can also further leverage existing alliances, that is, African Union or European Union, to share knowledge among themselves and to coordinate policy statements on agenda items (which they already do). The number of delegates on civil society delegations should also be capped, as long as their meaningful engagement in the proceedings—in line with the framework of engagement with non-state actors—can be ensured.

The size of delegations also raises questions of fairness. The WHA formally operates on a one country, one vote system. Yet, larger delegations have an advantage because they can cover more meetings and engage in more networking opportunities. If delegation sizes were capped, it may create a more level playing field. Moreover, the past decade has seen an increasing roll for health attachés who can do more of the day-to-day work throughout the year in Geneva and lay the groundwork for a smooth WHA.

In this context, fairness and equity can be difficult to define and implement in practice. Again, asking most delegates to watch from home is only fair if there is equitable access to reliable and secure internet. Large donor countries and non-donor countries have different ideas around what is a fair allocation of seats in the negotiation rooms. Also, if more work is shifted from the WHA to health attachés, then the global community needs to ensure that all countries have the resources to make this change in diplomatic practice.

Second, as laid out in Article 14 of the WHO’s Constitution, the WHA can take place in any country. Holding the WHA outside of Geneva presents many logistical challenges and I do not suggest it is ideal, only that it is possible. The 2nd WHA was held in Rome (1949), the 8th WHA in Mexico City (1955) and the 11th in Minneapolis (1958). COVID-19 is a global pandemic, affecting nearly all countries. However, if there were a public health emergency contained to Europe or Geneva, it would still be possible to hold the WHA in a safer location (although this should have been approved by the previous WHA and the Executive Board). Beyond the current challenge, concerns have also been raised over the suitability of the Palais de Nations, especially if the size of the event continues to increase.^5^

The length and frequency of the Assembly can also be reconsidered. Without a constitutional change, the WHA needs to take place annually as stated in Article 13. At the 3rd WHA in 1950, Scandinavian countries, concerned over the costs and burden of work on the Secretariat and Member States, proposed that the WHA be held biennially. This included idea that the WHA and regional committees could be held in alternating years, reducing workload of delegations who would otherwise attend both^6^ This was never implemented, but it is an idea worth reconsidering, especially in light of the climate impact. Similarly, some agenda items could be pushed to regional committees. The length of the WHA has also fluctuated over the past 73 years, with the 1st WHA lasting a full month, although there was much to discuss as the organisation was in its infancy.

The first priority at the virtual WHA is to handle the COVID-19 crisis. The second priority is to ensure that all the other health issues that were on the agenda are not forgotten; to this end, hopefully the 73rd WHA can reconvene in a resumed session later in 2020. The third priority is to take stock of what we need from the Assembly, both its formal and informal functions. What is the ideal balance between formal proceedings, technical discussions, side events and serendipitous meetings in the lounges and how can this balance feasibly change in future WHAs? And, taking into consideration fairness and equity, what tasks can be shifted online or to health attachés? The issue of maintaining the WHA’s essence while addressing 21st century challenges is something for the global health community to consider this May.

## Data Availability

There are no data in this work.
